# Editorial: Lipid metabolism in metabolic dysfunction- associated steatotic liver disease

**DOI:** 10.3389/fmed.2025.1584932

**Published:** 2025-03-27

**Authors:** Sheetalnath Rooge, Nirajan Shrestha

**Affiliations:** ^1^Liver Center, Division of Gastroenterology and Hepatology, Department of Internal Medicine, Kansas University Medical Center, Kansas, KS, United States; ^2^Liver Center, Division of Gastroenterology, Massachusetts General Hospital, Harvard Medical School, Boston, MA, United States

**Keywords:** MASLD, lipid metabolism, liver fibrosis, free FA (FFA), MASH, obesity, metabolic disease

The Metabolic dysfunction Associated Steatotic Liver Disease (MASLD) is the most common cause of chronic liver disease. A meta-analysis published in 2016 reported the global prevalence of MASLD is 25% and it was the leading cause of chronic liver disease then (1). A recent systematic review showed the global burden of MASLD has increased to an estimate of about 38% (2). In the past 3 decades the prevalence of MASLD has steadily increased by 50% and it continues to grow (3). The overall prevalence of MASLD in men (39.7%) is significantly higher than women (25.6%). The estimates of incidence rates of MASLD were 46.9 cases per 1,000 person-year, with 70.8 cases per 1,000 person-year in men and 29.6 cases in 1,000 person-year in women (4). An important subset of MASLD is metabolic dysfunction-associated steatohepatitis (MASH) which is major cause of disease progression into advanced fibrosis, cirrhosis and hepatocellular carcinoma. Here we are adopting the new nomenclature and referring to previous publications of NALFD and NASH with MASLD and MASH respectively (5).

Liver is the most essential organ that metabolizes nutrients. It plays crucial role in maintaining optimum levels of nutrients in the blood in fast and fed state. In the liver, lipid metabolism is regulated during uptake of lipids from circulation, *de novo* lipogenesis, oxidation, and export of lipids. Dysregulation of lipid metabolism in the liver leads to excess deposition of lipids in the hepatocytes that eventually lead to development of steatosis in the liver. Accumulation of steatosis in the liver is a characteristic of metabolic dysfunction-associated liver disease.

The well studied reasons for impaired metabolism in the liver are high fructose containing diet, sedentary lifestyle, high BMI, diabetes, heart diseases, and genetic risk factors. Insulin resistance is a pathological condition known to cause diabetes and is also among the factors responsible for development of MASLD. Cao et al. found a positive correlation between insulin resistance and development of MASLD in patients with metabolic syndrome. Genetic risk factors do play an important pathological role in the development of MASLD, a known example is the variations in the gene PNPLA3 (6). Other than this, various genes and transcription factors involved in lipid metabolism are known to play a causative role in MASLD development. A review published by Rao et al. highlights the role of lipid metabolism in MASLD with special emphasis on the mechanism of SREBP1c, ChREBP and PPARs in the *de novo* lipogenesis. Sexual dimorphism has a significant role in the pathophysiology of MASLD development. Prevalence of MASLD is higher in men than woman (7). A brief report published by Thomas and Njoku showed the sexual dimorphism in expression of hepatic PPAR alpha and CYP4a12a and its inverse association in development of drug induced MASH.

Obesity, Insulin resistance, diabetes, and metabolic disease are the leading causes of increased free fatty acids (FFA) in blood. The circulating FFAs in the blood reach the liver where hepatocytes take them up in the majority and other immune cells. Excess uptake and accumulation of lipid molecules impair the capacity of hepatocytes to oxidize these lipid particles. Increased blood sugar levels and associated hyperinsulinemia lead to an increase in *de novo* lipogenesis enhancing the accumulation of lipids in the hepatocytes. Lipid accumulation also increases due to impaired or inefficient lipid exports from the hepatocytes. Circulating lipids such as FFAs, VLDL, LDL, and HDL molecules in the blood play a significant role in the development of MASLD. A study published by Xuan et al. reported the positive correlation between the ratio of remnant cholesterol (RC) to high density lipoprotein associated cholesterol (HDL-C) with independent increased risk of MASLD development and increased severity. The impaired lipid profile is known to cause cardiovascular disorders. Both MASLD and cardiovascular diseases are interlinked a study published by Miao et al. concluded that increased fatty liver index positively correlate with increased susceptibility of coronary heart disease in adult Chinese population.

The accumulation of excess lipids in the hepatocytes for prolonged period causes inflammation. The inflammasome activation can also cause inflammation that is responsible for pyroptosis, apoptosis, and ferroptosis. A recent article published by Wang et al. reviewed the present literature about the role of ferroptosis in development and prognosis of MASLD. These inflammatory conditions lead to the activation of various inflammatory pathways including the activation of NLRP3 as liver tires to get rid of these inflammatory cells by apoptosis. Liver cells produce varied amounts of extracellular vesicles (EVs) with different properties. EVs play important roles in cellular communication that regulate inflammation of hepatocytes (8). Tamimi et al. reviewed the role of extracellular vesicles in development of MASLD suggesting that these pathways play important role in the pathophysiology of MASLD.

In the past decade sodium-glucose co-transporter 2 (SGLT2) inhibitors and glucagon-like peptide-1 (GLP1) agonists are explored for the treatment of cardiometabolic treatment and type 2 diabetes (2) were also evaluated for the MASLD. In 2024 FDA approved the resmetirom therapy for the treatment of MASLD. It is a thyroid hormone receptor beta agonist (9). Even though many traditional treatment modalities that are currently in use are evaluated for the effectiveness in treating MASLD. A metanalysis and review published by Mou et al. reports the efficacy and safety of Dachaihu decoction in treating the MASLD.

In summary the articles published in this special topic provide various aspects of MASLD, ranging from role of lipids to available therapeutic intervention and their comparison with traditional treatment options. The role of lipid metabolism in the development of metabolic dysfunction-associated liver disease is crucial and demands more research.
